# DDRprot: a database of DNA damage response-related proteins

**DOI:** 10.1093/database/baw123

**Published:** 2016-08-25

**Authors:** Eduardo Andrés-León, Ildefonso Cases, Aida Arcas, Ana M. Rojas

**Affiliations:** 1 Computational Biology and Bioinformatics Group, Institute of Biomedicine of Seville, 41013 Sevilla, Spain; 2 Cell Movements in Development and Disease Lab, Instituto de Neurociencias (CSIC-UMH), 03550 Alicante, Spain

## Abstract

The DNA Damage Response (DDR) signalling network is an essential system that protects the genome’s integrity. The DDRprot database presented here is a resource that integrates manually curated information on the human DDR network and its sub-pathways. For each particular DDR protein, we present detailed information about its function. If involved in post-translational modifications (PTMs) with each other, we depict the position of the modified residue/s in the three-dimensional structures, when resolved structures are available for the proteins. All this information is linked to the original publication from where it was obtained. Phylogenetic information is also shown, including time of emergence and conservation across 47 selected species, family trees and sequence alignments of homologues. The DDRprot database can be queried by different criteria: pathways, species, evolutionary age or involvement in (PTM). Sequence searches using hidden Markov models can be also used.

**Database URL:** http://ddr.cbbio.es.

## Introduction

The DNA Damage Response (DDR) is a crucial signalling network that maintains the integrity of the genome (1, 2). This network is the final result of a complex assembly of different sub-networks, delicately integrated according to the type of damage inflicted on DNA. This response requires the action of different proteins acting in concert, and it is highly dynamic. Due to its essential role in combating genome instability and therefore, its central influence on disease (3, 4), DDR pathways have been used as therapeutic targets (5), with molecules like PARP (Poly ADP-Ribose polymerase) inhibitors (6, 7) or more recently ATR (Ataxia telangiectasia and Rad3 related) inhibitors (8–10) seen to have potentially beneficial effects. However, despite the importance of this DDR network, information about its components, their interactions and its evolution as well as emergence has rarely been compiled (11), perhaps with the exception of DNA repair for which some resources are available (http://sciencepark.mdanderson.org/labs/wood/DNA_Repair_Genes.html).

This prompted us to conduct a comprehensive analysis of the DDR network and its sub-pathways (27), completing and updating the work carried out previously (11). In this previous work, we collected curated information about human proteins involved in most of the pathways and classified them into four sub-networks to facilitate further studies: global repair; checkpoints; responses at replication forks; and response at double strand breaks. We also assigned them to one or more of four functional classes: ‘sensors’ that identify and locate the damage produced by chemical alterations or by strand breaks; ‘transducers’ that label damaged sites and trigger a localized signalling response; ‘mediators’ that activate checkpoints or serve as protein/protein interactions platforms; and ‘effectors’ that ultimately lead to death or repair (12). This four-tier classification has been proposed previously and it is widely employed in the DDR field (12–14). Being post-translational modifications (PTMs) a hallmark of DDR regulation, we manually collected evidences from the existing literature, including phosphorylations, by far the most studied modification, and other essential modifications that control the DDR including ubiquitination, sumoylation and acetylation (15, 16). While some relevant evolutionary studies have been carried out in the past decade (17, 18), few have focused on the network as a whole, with studies often restricted to just a few protein families or with particular emphasis on chromatin modulators (19) and repair enzymes, and often confronted with the additional difficulties due to the miss-assignation of orthology based on functional analogy, a common error in the field of cell biology that emerges when extracting information from the literature (20) and skews the interpretation of evolutionary data. To fill this void and overcome these problems, we assigned evolutionary ages to these proteins in order to infer when these pathways emerged, we scored the presence or absence of these proteins in model organisms, and computed probabilistic-based phylogenies (21). Among other findings, we highlighted the absence of entire modules within pathways in model organisms, and even complete pathways (21), indicating the existence of specific lineage expansions (22), as well as the poor conservation of important pathway modules that have been used as therapeutic targets (e.g., the BRCA1 [Breast cancer type 1 susceptibility protein] complex).

Since these data could be of interest to the scientific community working on DDR, we have created a structured database and the corresponding web interface to aid the exploration of this resource.

## Methods summary

The implementation of the DDRprot database was divided into two steps: (i) in the first step information was obtained for humans and their homologous proteins in 46 species, whereby the information from different sources is integrated into a relational database and a Perl script downloads; (ii) a user friendly web interface that displays the data in a graphical format that enables DDR proteins emergence during evolution to be visualized, as well as its function and the sites of PTM in 3D structures.

Protein sequences and other information where obtained from the Energy's Joint Genome Institute (http://genome.jgi-psf.org/, Assembly 1.0: *Ciona intestinalis, Capitella teleta, Emiliania huxleyi, Monosiga brevicollis. Branchiostoma floridae, Naegleria gruberi, Nematostella vectensis* and *Trichoplax adhaerens;* Assembly 1.1*: Physcomitrella patens subsp. Patens;* Assembly 2.0*: Chlamydomonas reinhardtii, Phaeodactylum tricornutum (strain CCAP 1055/1);* Assembly 4.1*: Xenopus tropicalis*), NCBI (RefSeq Release 68), Uniprot (release 2014_10), Ensembl (Ensembl 77), WormBase ParaSite(version 2.0). Available structures for human DDR proteins were obtained from Uniprot. Mapping of amino acid residues into structures is done using SIFTS (23) (https://www.ebi.ac.uk/pdbe/docs/sifts/). Detailed information on how orthologues where inferred [using Inparanoid (v4.1) (24)], gene age was calculated [using Count (25)] and trees where compiled [using MAFFT (Version 7) (26) and MrBayes (3.2.5) (27)], can be found at Ref. (21). Searches based on sequence similarity are performed using the phmmer program from HMMER3 (v3.1b2) (28)

The DDRprot database is a relational database developed in MYSQL (version 5.5.40). The front end is comprised of a website designed in PHP (version 5.3.10), CSS3 and with a JQuery JavaScript library, and for PDB structures visualization, it uses the BioJS (http://biojs.io/d/bio-pv) protein viewer at WebGL capable browsers and JSmol (http://wiki.jmol.org/index.php/JSmol) at others. The PTMs interactions are shown using an arc-plot inspired on Daniel Gaston’s ‘arcdiagram’ (https://github.com/gastonstat/arcdiagram) and use the D3 JavaScript library. Sequence alignments visualizations use the JavaScript Sequence Alignment Viewer (JSAV, https://github.com/AndrewCRMartin/JSAV).

## Database description

**Figure 1.**
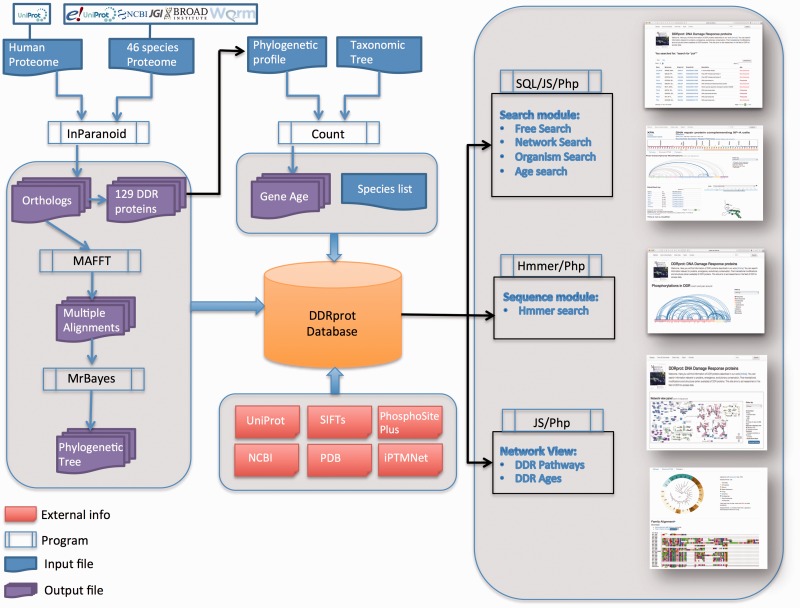
DDRprot Workflow. This diagram depicts the workflow of input/output data in DDRprot.

The DDRprot database contains comprehensive information about 129 human proteins involved in the DDR network. This set includes proteins demonstrated to be involved in DDR functions via accurate bibliographic information avoiding automatic extension, which in turn may lead to misleading function assignment. For each protein we provide a functional role, links to sequence databases, a list of synonyms, a list of the PTMs it undergoes and those that it exerts over other DDR components (within the DDR database). Links pointing to PhosphositePlus (http://www.phosphosite.org) and iPTMnet (http://research.bioinformatics.udel.edu/iptmnet/) are provided for DDR proteins that are modified by other proteins in alternative contexts. Finally, we provide the list of known 3D structures of human DDR proteins in our database. Regarding evolutionary information, we provide conservation profiles and links to orthologues in 46 additional species that represent key points in evolutionary history. These profiles were used for the construction of protein sequence alignments, as well as phylogenetic trees, which are also provided in both text and graphic formats. All this information can be downloaded in three separate files: one SQL dump of the database (the database schema is also available), a fasta-format file containing all human DDR protein sequences and their orthologues in the 46 selected representative species, and a compressed directory containing for each protein family, the phylogenetic tree in text format and the multiple sequence alignment in both fasta and nexus formats. Commands to compute the phylogenetic tree with Mr. Bayes (27) are indicated inside the nexus format. A workflow diagram is presented (Figure 1).

## Web interface

### Main views

The DDRprot site contains detailed information about how to use the site, with a video help and documents, as well as contact information. These features can be found at the very top of every page, where an additional search tool is also included for fast access. The site is accessible to different devices and at different resolutions.

**Figure 2.**
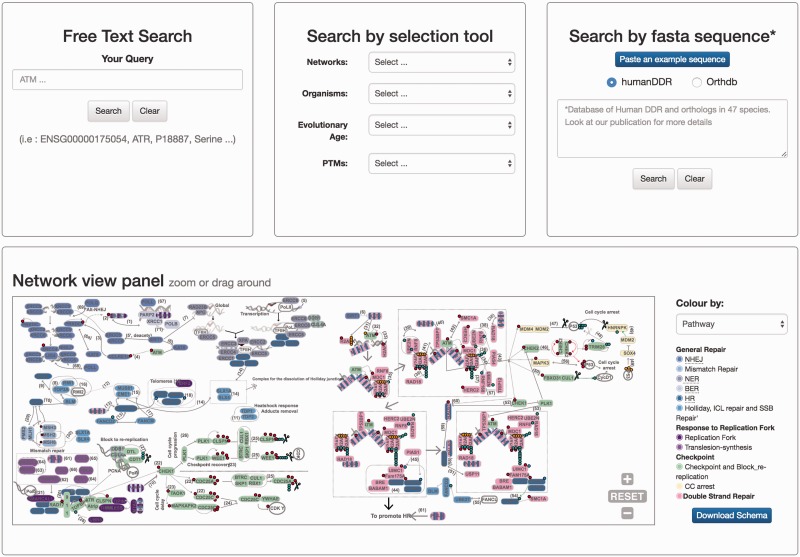
Homepage view. This picture shows a partial view of the homepage depicting available searching modules at DDRprot. From top to bottom and left to right, the ‘free text’ mode, the ‘selection tool’ by type of network, type of organism, evolutionary age of proteins, and post-translational modifications (PTMs). The ‘sequence search’ module uses phmmer from HMMER3, and searches can be restricted to the human proteins or human plus orthologues. The ‘search by clickable image’ module enables graphical searching, where proteins can be ordered by pathway or by evolutionary age. Numbers in the interactions are clickable and point to the PubMed reference describing the relationship. When a protein belongs to more than one pathway, this is indicated by vertical coloured bars. This schema is downloadable.

The home page is dominated by a clickable map of the network, the ‘Network view panel’ (Figure 2). This image can be zoomed and panned to the desired proteins/pathways. A colour-based scheme depicts the different sub-networks, facilitating their visual organization, which can be changed to evolutionary conservation. This map enhances the context-based exploration of the network (i.e. accounting for neighbouring proteins, etc.) while highlighting either its modular structure or its evolutionary past. It contains links to the bibliographic references supporting all the interactions shown (numbers in the map), and clicking in protein nodes leads the user to the specific protein display page. The map can also be downloaded in ‘SVG’ (Scalable Vector Graphics) format.

**Figure 3.**
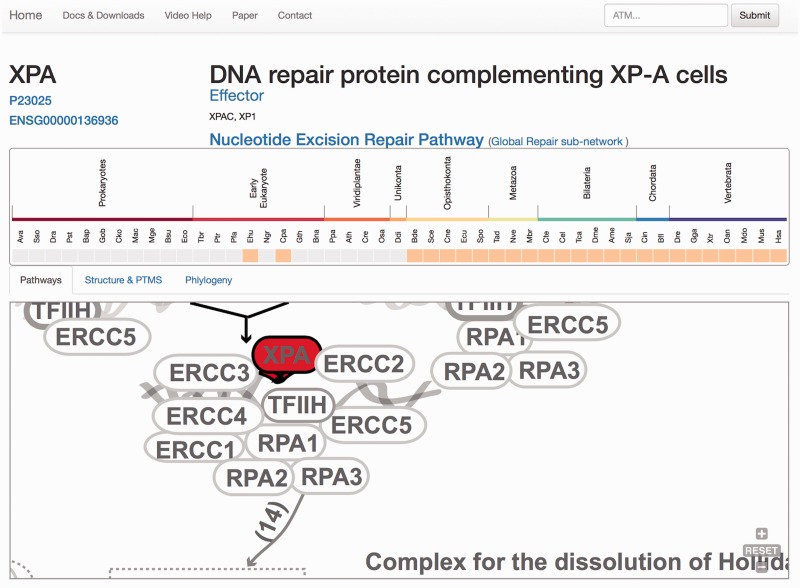
Protein results page, ‘Pathways tab’. The query is the protein XPA. A general description of the query protein is depicted with clickable links. The evolutionary emergence box shows the phylogenetic profile of the protein in 47 species, where orange squares indicate its presence and gray indicate absences (orthologue not found or missing). Species are named using a three-code naming convention (available in the help menu) although rolling over will display full names. The squares are clickable and point to the source of the protein. This emergence box appears constant in all the remaining tabs when selected. In the ‘Pathways’ tab, XPA, is highlighted in red and its location within the pathway is zoomed in, whereas the rest of the proteins are coloured in gray.

At the protein display page (Figure 3), the upper section contains a general description of the protein, synonyms, the network/s and the pathway/s with which it is associated, its functional assignations according to the four-tier classification (12). Links to Uniprot and Ensembl are also provided, allowing easy access to protein features and genomics context. Below, the pattern of protein presence/absence in the 46 representative species is presented, allowing a rapid perusal of evolutionary conservation and emergence (21).

**Figure 4.**
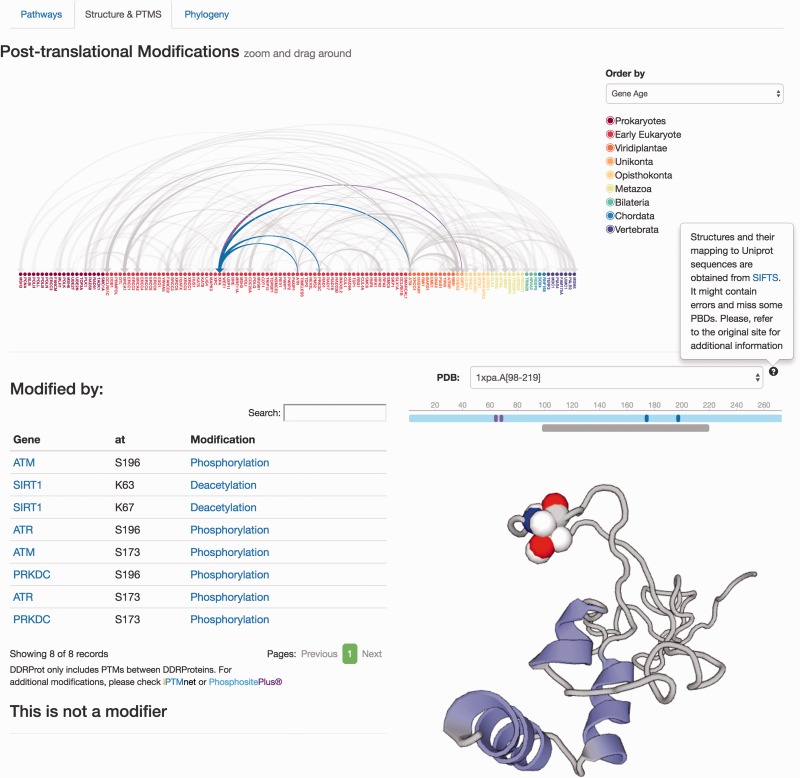
Protein results page, ‘Structure and PTMS’ tab. It provides information regarding Post-translational modifications described in the literature as well as the protein interaction map formalized as an arc-plot within DDR proteins. This map can be ordered by different criteria (gene name, gene age or network), and it will reorder dynamically when changed. The map is clickable and can be zoomed in/out. Links to PTM repositories are provided for the modified protein. If the protein has structural data, PDB structures are shown and PTMs are mapped to these structures if possible using SIFTS. A schematic representation of the protein over the structure indicates the length of the protein sequence (light blue line), the positions of the residues, which are modified (solid vertical bars), whereas the lower gray bar indicates the protein coverage of the selected structure. Clicking on the gray line will point to the PDB structure page. Menus at the left indicate position and type of modifications, and whether the protein is a modifier and/or is modified. Clicking in the modification type directs to the PubMed entry describing the modification.

The remaining information is distributed in different tabs, including the ‘Pathways’ tab that provides a zoomed version of the DDR clickable interaction map where a detailed view about the context and neighbouring proteins is shown. As all other visual maps in the DDRprot website, this detailed view can be downloaded as a ‘SVG’ file. The ‘Structures and PTMS’ tab (Figure 4) shows the repertoire of DDR proteins subjected to PTM within the context of the DDR (importantly, this information is extracted from the literature), as well as the precise residues or regions involved in the different modifications. It also provides structural coverage of the selected protein and its modified residues when possible. The interactions within proteins in DDRprot are shown using an arc-plot, where proteins can be ordered according to different criteria (gene age, gene name or pathway). Links pointing to PhosphositePlus and iPTMnet are provided to explore PTMs outside the DDR context.

**Figure 5.**
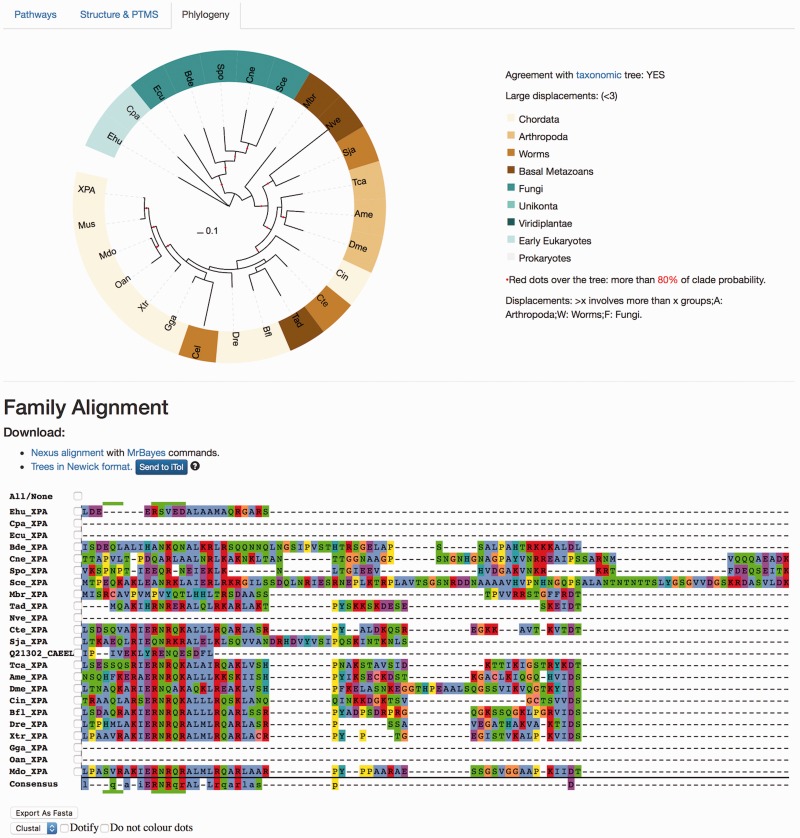
Protein results page, ‘Phylogeny’ tab. This module shows evolutionary information. It displays the consensus probabilistic-based phylogenetic tree of homologous proteins to the query protein. In the tree, the name of the protein (in our case, XPA) is shown for Homo sapiens, while the rest are named by our three-code letter species naming (available in the help section, the same found in the emergence tab) where the nodes are coloured according to the main groups widely used in the evolutionary field for visualization purposes. A species/taxonomy tree is provided also from this page, to check for agreement. The multiple sequence alignments displayed are the unprocessed alignments of homologous sequences as generated by MAFFT (see main text) and can also be exported as ‘fasta’. Both the ‘Nexus alignment with MrBayes commands’ and ‘Trees in Newick format’ files can be downloaded. The former includes an explicit definition of excluded regions and also includes MrBayes running commands to compute the tree, while the latter is the consensus tree in standard ‘Newick’. It is possible to send the tree to the iTOL site for customized visualization.

The ‘Phylogeny’ tab shows evolutionary information, where both multiple sequence alignments and phylogenetic trees are available and can be downloaded in different formats (Figure 5). The raw ‘fasta’ alignments of homologous proteins [MAFTT-generated using the slow and accurate version (26)] are also available to visualize and/or download. Those are the direct program outputs that can be obtained without further editing so that the user can visualize these and select regions of interest. The ‘Nexus alignment & MrBayes commands’ format contains the alignment used to draw up the phylogenetic trees and in each file, the commands used to run MrBayes (27) are also specified at the end of the alignment block. In addition to the ‘SVG’ graphics, it is also possible to download the consensus phylogenetic trees in the classic ‘Newick’ raw format, and also to directly upload the tree in iTOL site (29) to enable custom visualization.

### Querying the database

There are two ways to access the data in DDRprot, using different panels adapted to the distinct search strategies: free text search, or through a selection tool (Figure 2). The free text form provides a quick and flexible means to search specific proteins in our database using identifiers or keywords. With the selection tool users can search for proteins involved in a particular pathway or network, present in particular species, or with a given gene age.

**Figure 6.**
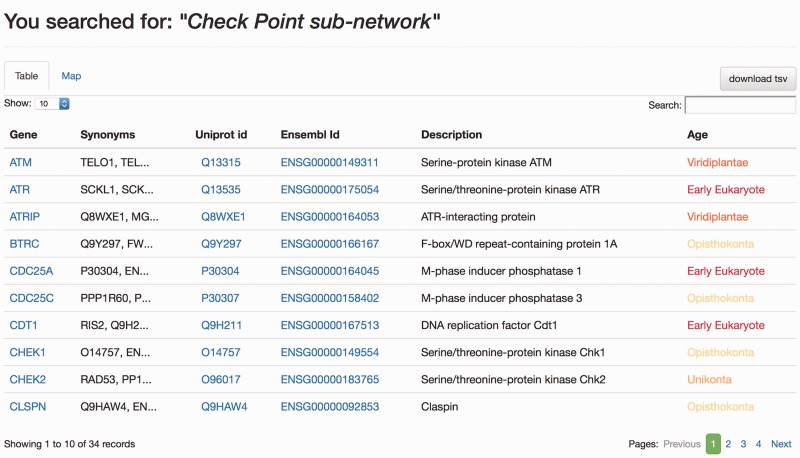
Results page, ‘Table’ view. The query was by ‘Check point sub-network’. The ‘Table’ tab shows the results in a tabular format, which can be ordered by any column. The user can select which information to download from the table. Clicking in gene names will direct the user to the Gene page results display.

**Figure 7.**
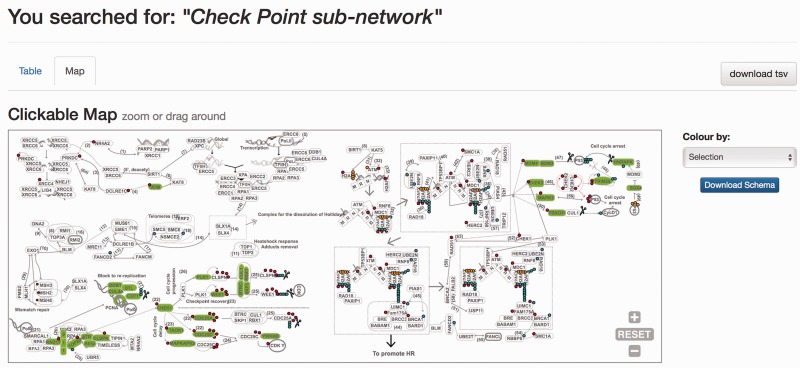
Results page. Results page, ‘Map’ view. The query was by ‘Check point sub-network’. The ‘Map’ tab depicts the proteins belonging to this query within the DDR network to provide context. The selection can be coloured by either pathway or by evolutionary age of the pathway. The schema is downloadable as SVG format file.

When the DDRprot is searched using these selection tools (i.e. pathways, species or evolutionary age), results are shown both in a clickable table (Figure 6) and in a downloadable map (Figure 7). In the ‘Map’ tab, it is possible to change the colouring pattern to highlight the precise pathways including the selected proteins or their evolutionary conservation. In the ‘Table’ tab, each row represents a protein where general information is provided. The set of proteins found can be downloaded as a tab-separated-values text file, and fields included in the file can be selected at will. Clicking in the ‘Gene’ identifier will direct the user to the protein display page.

Alternatively, the user can select to visualize a particular set of PTMs within the DDR proteins. In this case, the system will provide an interaction arc-plot. Genes in this plot are ordered by gene-name but can be dynamically re-ordered by different criteria (network or gene-age) highlighting the intra- or inter-class network of the interactions. The plot is clickable and checking on protein names will direct the user to the protein display page.

### Sequence searches

The DDRprot site enables the database to be searched based on sequence similarity (Figure 2). By pasting a fasta sequence file, users can find homologues of their query protein sequence. For the search, the DDRprot database implements a probabilistic tool based on hidden Markov models to identify similar sequences [phmmer from the HMMERV.3 suite (28)].

Users can search against all orthologous DDR proteins (including all species), or restrict the searches to human DDR proteins. In the latter case, results are shown in the standard tabular and graphical formats obtained with any other search methods, but with the addition of the HMMER search scores and *E*-values (note that *E*-values in these searches are inflated due to the small database size). If the user selects the orthologues set, the results table also contains links to the original databases, as well as to the orthologous human gene page at DDRprot. In both cases, results are shown in both tabular and map formats (Figures 5 and 6). In addition, the user can download the original HMMER output in text format.

## Discussion

The DDR is a very complex network that is comprised of several pathways, each of which is involved in much cross-talk both within the network and with other signalling systems. One of the main limitations when studying the DDR is the absence of a comprehensive set of proteins identified by manual curation, which constitutes a trustable *bona fide* DDR catalogue. Along these lines, we have established a dataset of human proteins (21) and although such information was available in the literature, its fragmented nature made exploring these data difficult. For this reason, we created a web-based resource that aims to be useful and usable.

We believe that this resource will be very useful in several ways. It enables the DDR network to be explored in a graphic manner, where a rational classification of its sub-networks allows particular pathways to be easily extracted. This is potentially useful to design *ad-hoc* libraries in order to guide directed screenings of certain protein subsets (e.g. repair proteins, effectors or older, more conserved proteins). In this sense, it also allows orthologues in other organisms to be identified that could be useful as models. Although other DDR-related proteins, mainly paralogues, were not included (i.e. including paralogues of certain proteins would affect the results of emergence analyses) we believe the selected core protein set represents fairly the DDR network as they are experimentally confirmed. Other proteins, like P53 (a transcriptional regulator also involved in many other events) were excluded in purpose, to avoid the inclusion of noise (although the protein is represented in the schema to provide context) as the main focus of our work is to establish a well-curated dataset of DDR proteins. We did not include proteins extended by network analyses, as the focus was to keep a trustable literature-based and manually curated core set of proteins.

Multiple sequence alignments of homologues are drawn up using state-of-the-art methods (30) and as generated, they can be focused on particular regions that might be critical in the design of effector molecules. In addition, we indicate the regions that are excluded/included in the computation of the phylogenies in the ‘nexus’ format versions of the same alignments, along with the corresponding commands to run the phylogenetic analyses in MrBayes (27). Finally, information about PTMs within the context of structural data can also be of great help in structure/function studies when assessing regions of therapeutic interest. It is noteworthy that all the data included in the database are linked to its source publication in PubMed, making it easy to retrieve detailed information.

On top of the tools described above, it is also possible to search our database using sequences generated in-house, for which we have included a sequence search module based on HMMER that uses our custom databases (of small size) enabling for a quick search of custom sequences. The new version of HMMER (28) enables domains to be confidently identified reasonably quickly, as well as identifying orthologous proteins. Future improvements of the database will include updating the information available in literature on these proteins, to include protein domains assignations, to enhance the searching module using HMMER for larger queries, and to include any relevant pharmacological data.

To summarize, the DDRprot database is the first database devoted to a set of manually curated DNA Damage Response proteins. It is presented within an evolutionary framework that allows many features to be explored and as such, we believe it will be very useful to researchers in the field.
